# Whole-Genome Sequences of Six Listeria monocytogenes Strains Isolated from Food

**DOI:** 10.1128/MRA.01036-18

**Published:** 2018-10-11

**Authors:** Jule Anna Horlbog, Hyein Jang, Gopal Gopinath, Roger Stephan, Claudia Guldimann

**Affiliations:** a Institute for Food Safety and Hygiene, Vetsuisse Faculty, University of Zürich, Zürich, Switzerland; b Center of Food Safety and Applied Nutrition, U.S. Food and Drug Administration, Laurel, Maryland, USA; University of Delaware

## Abstract

Here, we report the whole-genome sequences of six Listeria monocytogenes strains isolated from meat and milk products in Switzerland. All of these strains carry premature stop codons or amino acid deletions in *inlA*.

## ANNOUNCEMENT

Listeria monocytogenes is a Gram-positive foodborne pathogen and the causative agent of listeriosis, which poses a significant public health risk because of its high case fatality rate, ranging from 15 to 30 deaths per 100 cases (1–4).

We have sequenced six strains of L. monocytogenes obtained from various foods between 2011 and 2014. These strains were isolated as described by Ebner et al. (5) and sent to the National Reference Center for Enteropathogenic Bacteria and Listeria (NENT) in Switzerland. PCR-based multilocus sequence typing (MLST) revealed that the strains are evenly distributed among lineages I and II; three isolates belong to clonal complex 6 (CC6), two isolates to CC9, and one isolate to CC121 (Table 1). Three of the strains were identified as serotype 4b, and two of the other three strains were serotyped as 1/2c. The last strain possessed the 1/2b serotypic determinant. All strains were stored at −80°C in 20% glycerol as frozen stocks. To obtain DNA, the stocks were streaked onto brain heart infusion (BHI) agar plates (Oxoid, Pratteln, Switzerland) and incubated overnight at 37°C. The following day, a liquid culture of a single colony from this plate was grown in BHI overnight at 37°C. Genomic DNA was extracted using the DNA blood and tissue kit (Qiagen, Hombrechtikon, Switzerland) and prepared for sequencing with a Nextera XT DNA library preparation kit (Illumina, San Diego, CA, USA). The samples were run in either 500 or 600 cycles of paired-end reads on a MiSeq platform (Illumina), and an average of 1,984,313 reads per strain were obtained. FASTQ data sets generated from the MiSeq platform runs were trimmed and *de novo* assembled using CLC Genomics Workbench version 9.0 (CLC bio, Aarhus, Denmark). Quality filtering and trimming of adapters were performed using Trimmomatic with default settings (6). The assemblies were annotated and identified through the NCBI Prokaryotic Genome Annotation Pipeline (7). As shown in Table 1, the genomes were found to have a length between 2,919,305 and 3,055,534 bp (average, 2,981,293 bp), 19 to 35 contigs, and between 2,891 and 3,049 (average, 2,964) coding sequences (CDS), with an average coverage depth of 146.2-fold.

**TABLE 1:** Overview of the strains examined in this study

Strain	Lineage	Sequence type	Clonal complex	Serotype	Isolation source	Yr of isolation	Genome size (bp)	No. of contigs	No. of CDS	*inlA* characteristic^*a*^	GenBank accession no.
N11-1848	II	9	CC9	1/2c	Meat	2011	3,036,227	33	3,024	PMSC	QEMB00000000
N12-0460	I	6	CC6	4b	Meat	2012	2,919,305	19	2,891	Deletion	QEMA00000000
N13-0703	I	6	CC6	4b	Milk	2013	2,927,591	20	2,898	Deletion	QELZ00000000
N13-0836	II	121	CC121	1/2b	Meat	2013	3,055,534	35	3,049	PMSC	QELY00000000
N13-1184	I	6	CC6	4b	Meat	2013	2,924,674	21	2,907	Deletion	QELX00000000
N14-0261	II	9	CC9	1/2c	Meat	2014	3,024,428	33	3,014	PMSC	QELW00000000

a PMSC, premature stop codon.

The BIGSdb database for Listeria (http://bigsdb.pasteur.fr/listeria) (8) verified our earlier PCR-based MLST and sequence type classifications of the strains. An analysis of the major virulence genes revealed that all Listeria pathogenicity island 1 (LIPI-1)-associated genes (*prfA, plcA, hly, mpl, actA,* and *plcB*) were present and are flanked by the following conserved housekeeping genes: *prs*, a phosphoribosyl synthetase gene, and *ldh*, a lactate dehydrogenase gene, as described by Vázquez-Boland et al. (9). None of the known prfA* mutations, which lead to the constitutive activity of PrfA, a major transcriptional regulator of virulence genes (10), were present in any of the sequenced strains.

Three of the strains (N11-1848, N13-0836, and N14-0261) carried premature stop codons (PMSC) in the *inlA* gene, which codes for a protein that interacts with host cell receptors. The stop codons occurred at different nucleotide positions (positions 2065 [N11-1848], 1486 [N13-0836], and 1742 [N14-0261]). The other three strains all carried the same three amino acid deletions in *inlA* at nucleotide position 2221.

### Data availability.

All sequences have been published in GenBank under accession no. QEMB00000000 (N11-1848), QEMA00000000 (N12-0460), QELZ00000000 (N13-0703), QELY00000000 (N13-0836), QELX00000000 (N13-1184), and QELW00000000 (N14-0261). The raw reads were deposited in the Sequence Read Archive (SRA) under accession no. SRS3471743 (N11-1848), SRS3471742 (N12-0460), SRS3471740 (N13-0703), SRS3471738 (N13-0836), SRS3471739 (N13-1184), and SRS3471737 (N14-0261).
